# Treatment outcome of patients with comorbid type 1 diabetes and eating disorders

**DOI:** 10.1186/1471-244X-14-140

**Published:** 2014-05-16

**Authors:** Nuria Custal, Jon Arcelus, Zaida Agüera, Francesca I Bove, Jackie Wales, Roser Granero, Susana Jiménez-Murcia, Isabel Sánchez, Nadine Riesco, Pino Alonso, José M Crespo, Nuria Virgili, Jose M Menchón, Fernando Fernandez-Aranda

**Affiliations:** 1 Department of Psychiatry, University Hospital of Bellvitge-IDIBELL, Barcelona, Spain; 2 Leicester Eating Disorder Service, Bennion Centre, Leicester Glenfield Hospital, Leicester, UK; 3 Loughborough University Centre for Research into Eating Disorders, Loughborough University, Loughborough, UK; 4 Ciber Fisiopatologia Obesidad y Nutrición (CIBERObn), Instituto Salud Carlos III, Barcelona, Spain; 5 Departament de Psicobiologia i Metodologia, Universitat Autònoma de Barcelona, Barcelona, Spain; 6 Departament of Clinical Sciences, School of Medicine, University of Barcelona, Barcelona, Spain; 7 Ciber Salud Mental (CIBERsam), Instituto Salud Carlos III, Barcelona, Spain; 8 Endocrinology and Clinical Nutrition Unit, University Hospital of Bellvitge- IDIBELL, Barcelona, Spain

**Keywords:** Dropout, Eating disorders, Personality, Psychopathology, Treatment outcome, Type 1 Diabetes Mellitus

## Abstract

**Background:**

Co-morbidity between Type 1 Diabetes Mellitus (T1DM) and eating disorders (ED) has been previously described; however the effect of this illness on the outcomes for conventional ED treatments has not been previously investigated. This study aims to compare clinical, psychopathological and personality features between two samples of ED individuals: those with comorbid T1DM and those without (No-DM); and to identify differences in treatment outcomes between the groups.

**Methods:**

This study compares treatment outcome, dropouts, ED psychopathology and personality characteristics for 20 individuals with ED and T1DM and 20 ED patients without diabetes, matched for diagnostic and treatment type.

**Results:**

The study found higher dropout rates from therapy in individuals with T1DM and worse treatment outcome in spite of having no significant differences in eating disorder psychopathology, although individuals with T1DM report misusing insulin.

**Conclusions:**

The low levels of motivation to change, and insulin abuse in T1DM patients, may suggest that treatment for patients with ED and T1DM should consider the individual’s personality and role of insulin abuse when determining the appropriate intervention.

## Background

The onset of Type I Diabetes Mellitus (T1DM) is usually associated with initial weight loss but insulin initiation and correct compliance can increase body mass index [BMI (Kg/m^2^)]. Research has found that T1DM is associated with high BMI, especially in young females [1]. Some of the weight increase may be associated with binge episodes triggered by hunger due to hypoglycaemia. Thus, T1DM individuals may be a susceptible population to develop overconcern about weight and shape, body dissatisfaction, food preoccupation, active dietary restraint and even disturbed eating behaviors. This may explain why T1DM individuals are at risk of developing certain eating disorders (ED), particularly bulimia nervosa [2].

ED in T1DM individuals can be manifested by weight-control practices that include fasting, vomiting, laxative and diuretic abuse, but also intentional insulin omission or under-dosing causing weight loss [2-4]. ED in T1DM has been associated with impaired metabolic control, more frequent episodes of ketoacidosis and an earlier-than-expected onset of microvascular complications [5].

In spite of this, current treatments for individuals with ED do not vary depending on whether the person suffers from T1DM or not, although the clinical and personality characteristics may differ. Research examining the personality characteristics and temperament profile in the field of eating disorders has identified that certain eating disorders, such as bulimia nervosa (BN) purging type, are included in the maladaptive profile, particularly related to impulsivity, which will be associated with high dropouts. In addition restrictive symptomatology is overrepresented in the adaptive profile [6], however this study has included eating disorder independent of their medical comorbidity, such as diabetes. With this in mind this study aims: a) to compare clinical, psychopathological and personality features between two samples of ED individuals: those with comorbid T1DM (T1DM) and those without (No-DM); and b) to identify differences in treatment outcomes between the groups.

## Methods

### Participants

From an initial sample of 1887 patients diagnosed with ED according to the DSM-IV [7] at the Eating Disorder Unit of the Bellvitge Hospital (Barcelona, Spain) between April 1999 and June 2012, 20 subjects with comorbid T1DM were identified (1.06% of total ED cases). Subjects suffering from T1DM were matched with 20 individuals without diabetes from the same pool of ED patients for socio-demographic variables (age, marital status, catchment area and education level), age of onset and diagnosis. Exclusion criteria were: (a) patients aged <18 years old; (b) males, as their number was too small for meaningful comparisons; (c) a T1DM diagnosis of less than 6 months before inclusion into the study; (d) patients diagnosed with other type of diabetes. In order to aid the interpretation of the results and make it relevant to the current DSM-5 diagnosis, the EDNOS category will be divided into patients with sub-threshold AN (who fulfill the diagnosis of AN under the new DSM-5), patients with sub-threshold BN (who fulfill the diagnosis of BN under the new DSM-5) and patients with “Pure” EDNOS.

### Assessment

Clinical information and diagnosis was obtained through a semi-structured interview conducted by experienced psychologists and psychiatrists. Patients completed a battery of psychological questionnaires, which included the Temperament and Character Inventory–revised (TCI-R) [8] and the Eating Disorders Inventory-2 (EDI-2) [9]. The motivational stage of change was assessed through a visual analogue scale, described elsewhere [10]. Anthropometric measures (weight and height), glycaemic control and insulin adherence were assessed during a scheduled face-to-face medical visit. For T1DM patients, the glycated hemoglobin (HbA1c) measured nearest to the interview was used as an indicator of glycaemic control. Insulin misuse was identified if patients admitted to manipulating or omitting their insulin dose as a purging method.

Following assessment and diagnosis, patients received treatment as usual: 16 sessions of cognitive-behavioural therapy (CBT) outpatient treatment for those with BN, binge eating disorder (BED) and eating disorder not otherwise specified (EDNOS), and 3 months day hospitalization for anorexia nervosa (AN) patients [11,12]. Patients were re-evaluated at the end of their treatment and categorized as: total remission, partial remission and no remission defined as per previous studies [12] and voluntary treatment discontinuation (dropouts). The Ethics Committee of our institution (Ethics Committee of Clinical Research of the University Hospital of Bellvitge) approved the study. Written informed consent was obtained from all participants.

### Statistical analysis

All statistical analyses were conducted using the Statistical Package for the Social Sciences, SPSS version 20.0 for Windows. T-TEST procedures compared quantitative clinical outcomes between ED patients with and without diabetes, and binary logistic regressions compared categorical clinical outcomes. Survival analysis modeled time to therapy dropout, Breslow chi-square test compared survival cumulative functions for patients with and without diabetes. All the models were adjusted for the variables of BMI and ED diagnostic subtype. A p-value of <0.05 was considered statistically significant. Effect-size analyses were performed using Cohen’s d, which represents the mean difference score between two groups. A Cohen’s d score <0’3 was considered small, >0’3 and <0’8 medium and >0’8 large.

## Results

No statistical differences were found between the two clinical groups for any socio-demographic variable. Mean age for T1DM patients was 25.3 years (SD = 8.0) and for No-DM 28.0 (SD = 8.4). Age of onset of ED was 19.5 years (SD = 7.4) for T1DM and 19.4 (SD = 7.5) for No-DM. ED diagnostic distribution was: 10.0% “AN and sub-threshold AN”, 25.0% “BN and sub-threshold BN”, 10% BED and 55% “pure” EDNOS (the same distribution for the both groups, for T1DM and for No-DM, as they were matched on this variable). For T1DM patients, the mean age of the diabetes diagnosis was 15.1 years (SD = 6.2, interquartile range from 10.5 to 17 years) and the mean duration of the illness was 10.3 years (SD = 8.2, interquartile range from 3 to 15 years). In this group of patients, mean HbA1c was 9.94% (SD = 3.00) (normal range 4.5-6%) at baseline.

T1DM used insulin manipulation to control weight as most of them (n = 18, 90%) acknowledged skipping or reducing insulin doses. Prior ED onset, poor self-monitoring of glycaemic control characterized these T1DM subjects. Except for insulin misuse, there were no statistical differences between the two clinical groups for any other eating disorder related behaviour and psychopathology (see Table 1). No differences were found in the frequency of bingeing or diuretic abuse. However, although not statistically significant, use of vomiting and laxative abuse was more prevalent in the No-DM group. Those with T1DM were significantly less likely, than No-DM, to self-harm (p < .012; d:1.03), have suicidal ideation (p < .030; d:0.80) and suicidal behaviour (p < .039; d:0.79) (see Additional file 1: Table S1).

**Table 1:** Comparison for clinical, motivational and personality traits at baseline between the two clinical groups (N = 40)

	**Mean – standard deviation**				**Mean comparisons and Cohen’s-d**				
	**No-DM**		**With T1DM**		***p***	**Mean diff.**	**95% CI (mean differ.)**		**|** ***d |***
	**(*n*** **= 20)**		**(*n*** **= 20)**						
Binges per week	4.10	6.46	3.71	6.98	.860	−0.39	−4.88	4.09	0.06
Vomits per week	4.80	9.43	1.65	3.77	.205	−3.15	−8.11	1.80	0.44
Laxatives per week	5.20	15.71	0.82	3.40	.269	−4.38	−12.28	3.53	0.39
Diuretics per week	1.05	4.70	0.44	1.75	.625	−0.61	−3.13	1.91	0.17
Body mass index (kg/m^2^)	22.59	6.23	23.34	6.35	.722	0.75	−3.51	5.02	0.12
Perceived Intensity of ED	5.85	2.03	4.35	2.11	.028*	−1.50	−2.83	−0.17	0.72*
Need for treatment	5.90	2.40	5.30	2.41	.435	−0.60	−2.14	0.94	0.25
Impairment	5.50	2.26	3.65	2.66	.023*	−1.85	−3.43	−0.27	0.75*
Worry (self)	6.45	1.99	5.55	2.96	.266	−0.90	−2.52	0.72	0.36
EDI: Drive for thinness	13.11	6.00	12.15	5.40	.604	−0.96	−4.66	2.75	0.17
EDI: Body dissatisfaction	15.47	7.81	15.45	8.78	.993	−0.02	−5.42	5.38	0.00
EDI: Interoceptive awareness	14.11	7.74	9.50	6.82	.056	−4.61	−9.33	0.12	0.63*
EDI: Bulimia	5.58	5.90	5.70	5.70	.948	0.12	−3.64	3.89	0.02
EDI: Interpersonal distrust	6.68	3.51	5.75	4.66	.486	−0.93	−3.62	1.75	0.23
EDI: Ineffectiveness	11.21	7.07	10.90	7.91	.898	−0.31	−5.19	4.56	0.04
EDI: Maturity fears	6.74	5.59	8.75	5.22	.252	2.01	−1.49	5.52	0.37
EDI: Perfectionism	6.21	3.60	5.45	5.22	.601	−0.76	−3.68	2.16	0.17
EDI: Impulse regulation	7.32	4.66	7.55	7.07	.904	0.23	−3.67	4.14	0.04
EDI: Asceticism	6.63	4.18	6.60	4.31	.982	−0.03	−2.79	2.72	0.01
EDI: Social insecurity	8.42	4.26	8.95	6.39	.764	0.53	−3.01	4.07	0.10
EDI: Total score	101.47	39.10	96.75	48.80	.741	−4.72	−33.51	24.06	0.11
TCI-R: Novelty seeking	103.05	12.78	101.79	16.65	.795	−1.26	−11.03	8.50	0.09
TCI-R: Harm avoidance	118.32	15.11	115.42	21.43	.633	−2.89	−15.10	9.31	0.16
TCI-R: Reward dependence	105.32	12.52	101.84	16.94	.477	−3.47	−13.27	6.33	0.23
TCI-R: Persistence	119.47	22.68	101.16	29.31	.038*	−18.32	−35.56	−1.07	0.70*
TCI-R: Self-directedness	114.37	19.82	116.47	23.78	.769	2.11	−12.30	16.51	0.10
TCI-R: Cooperativeness	136.16	13.31	136.79	12.83	.882	0.63	−7.97	9.23	0.05
TCI-R: Self-Transcendence	68.95	14.50	65.84	12.16	.479	−3.11	−11.91	5.70	0.23

*Bold: significant comparison (.05 level). *Bold: moderate to high effect size (|*d|* ≥ 0.5).

T1DM group scored significantly lower on motivation to change and level of consciousness about their ED (less self-perceived intensity of the ED and less perceived impairment). The only personality trait with a significant difference was persistence, which showed lower scores in T1DM (participants were characterized by low perseverance, instability, giving up easily when confronted with frustration and with low accomplishment levels) than No-DM, and were lower than normative population scores [13].

### Treatment outcome

**Figure 1:**
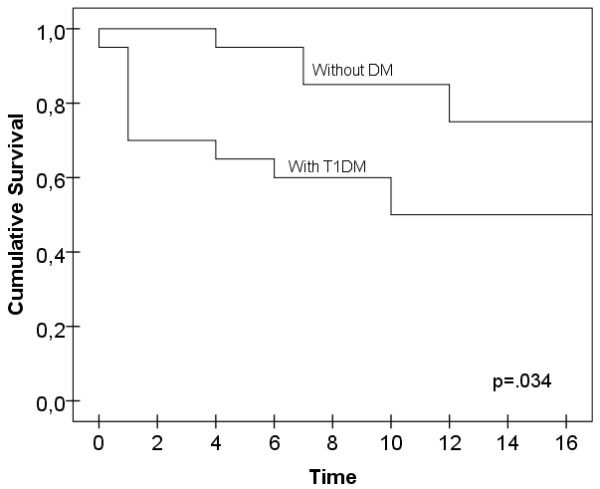
Cumulative survival.

Three quarters of No-DM group (n = 15) showed partial or full remission when compared to 50% (n = 10) of T1DM group (see Additional file 1: Tables S2-S3). The dropout number was higher among T1DM patients when compared to No-DM. Although the difference was not statistically significant, a Cohen’s d value of 0.53 shows at least 79% of the T1DM patients were higher dropout risks than the average non-diabetic patient. T1DM patients stopped treatment significantly earlier (χ^2^ = 4.50, df = 1, p = .034). From 50% of the patients that stopped treatment, 30% did so after the first session, 15% after the 4th, 5% after the 6th, and the rest after session 10. Patients without diabetes dropped out later on (see Figure 1). Dropout in T1DM patients was associated with lower motivation to change (both lower need of treatment, r = −.30, p < .05) and perceived intensity of ED (r = −.32, p < .05). No statistical differences, due to ED subtype, were found for the risk of dropout or partial-total remission in the No-DM patients (p = .284 and p = .523) and the T1DM group (p = .286 and p = .523) (Additional file 1: Table S2).

## Discussion

The aim of the study was to identify clinical differences and treatment outcome between individuals with eating disorders and T1DM and those without diabetes. Eating disorders, particularly BN has been previous associated to T1DM [2-5]. The association between both conditions is so clear that a new term, which is not yet a medically recognized condition, has recently been described in the literature, Diabulimia. This condition has been particularly described among the adolescent population, as weight management during this time of development for individuals with diabetes can be particularly difficult forcing them to restrict or omit insulin [14]. Our study confirms the misuse of insulin among this population. The lack of difference in purging behavior and the differences in self-harm and suicidal behaviour between the two groups may point towards the importance of analyzing the role of insulin misuse in this population. For example, the lower number of self-harm behaviours in patients with T1DM may indicate that patients with this condition may not require the use of self-harm in order to deal with emotions as they may use insulin misuse instead. It can be hypothesized that insulin omission, which leads to weight loss, is more closely connected to mood regulation and self-harm behaviour than to weight and shape issues, although this will need to be researched further. This could explain the difficulties that T1DM patients may have in stopping insulin abuse, as recently noted [4], and the poor motivation of these individuals as manifested by high number of treatment dropouts found in this study.

ED patients with T1DM were found to dropout more frequently and sooner than patients without diabetes. The study found that treatment outcome for T1DM patients was overall worse than for individuals without diabetes. Motivation levels and personality traits (low persistence and accomplishment levels), could explain these results, as suggested in studies with DM patients [15]. The fact that individuals with T1DM present with low persistence which is associated to low frustration tolerance and low perseverance may explain the high levels of dropouts and the poor outcomes found among these individuals. That and will suggest the need for interventions to be modified for this population and for diabetologies to be informed about the risk of poor outcome that patients with both conditions present. The lack of motivation for change found among individuals with T1DM may also be a reflection of the low levels of consciousness regarding the illness, the low perceived intensity and low perceived impairment which suggests the importance of working using Motivational Enhancement interventions with individuals with T1DM and ED.

Although there is clear evidence that T1DM are particularly associated with BN [2,5], this study has included all type of eating disorders. Future studies may want to focus in exploring the outcome of patients with T1DM and BN only. Moreover, future studies may also want to consider investigating whether the presence of comorbid diabetes impacts motivation to treatment per se. Although the study is limited by sample size, the patient’s reliability about self-reporting insulin underdosing and the possible influence of T1DM on ED questionnaires [16], the current study has addressed treatment response and dropout rates of CBT therapy across the ED patients with T1DM which, to our knowledge, has not been previously investigated.

## Conclusions

The low levels of motivation to change, and insulin abuse in T1DM patients, may suggest that treatment for patients with ED and T1DM should consider the individual’s personality and role of insulin abuse when determining the appropriate intervention.

## Abbreviations

AN: Anorexia nervosa; BED: Binge eating disorder; BMI: Body mass index; BN: Bulimia nervosa; CBT: Cognitive-behavioural therapy; DSM-IV: Diagnostic and Statistical Manual of Mental Disorders 4th edition; ED: Eating disorders; EDI-2: Eating Disorders Inventory-2; EDNOS: Eating disorder not otherwise specified; No-DM: Patients without diabetes mellitus; SD: Standard deviation; T1DM: Type I Diabetes Mellitus; TCI-R: Temperament and Character Inventory–revised.

## Competing interests

All authors declare that they have no conflicts of interest.

## Authors’ contributions

NC, JA, ZA, FB, SJM, PA and FFA designed the study. NC, ZA, FB, IS and NR collected the patient data. RG performed the statistical analyses. NC, JA and JW wrote the first draft of the manuscript. All authors commented on and approved the final manuscript.

## Authors’ information

Nuria Custal and Jon Arcelus shared first authorship.

## Pre-publication history

The pre-publication history for this paper can be accessed here:

http://www.biomedcentral.com/1471-244X/14/140/prepub

## Supplementary Material

Additional file 1: Table S1 Comparison for externalizing symptoms between the two clinical groups (N = 40). **Table S2.** Distribution for the outcomes baed on the ED subtype and the presence of the DM. **Table S3.** Comparison for therapy outcomes between ED with and without T1DM. Click here for file
