# Supplementary material for: Treatment outcome of patients with comorbid type 1 diabetes and eating disorders
Source: BMC Psychiatry. 2014 May 16;14:140. doi: 10.1186/1471-244X-14-140 (PMC4030033; doi:10.1186/1471-244X-14-140)
Supplement: Additional file 1: Table S1 — Comparison for externalizing symptoms between the two clinical groups (N = 40). Table S2. Distribution for the outcomes baed on the ED subtype and the presence of the DM. Table S3. Comparison for therapy outcomes between ED with and without T1DM. [file 1471-244X-14-140-S1.doc]

**Supplementary Tables (online)**

**Table S1**: Comparison for externalizing symptoms between the two clinical groups (N=40).

|  | Proportions | | Proportions comparisons | | | | |
| --- | --- | --- | --- | --- | --- | --- | --- |
|  | No-DM  n= 20 | T1DM  n=20 | *p* | OR | 95% CI  (OR) | | | *d |* |
| Self-harm behaviour | 55.0% | 11.8% | **.012*** | 0.11 | 0.02 | 0.61 | **1.03*** |
| Suicidal ideation | 60.0% | 23.5% | **.030*** | 0.21 | 0.05 | 0.86 | **0.80*** |
| Suicidal behaviour | 45.0% | 11.8% | **.039*** | 0.16 | 0.03 | 0.91 | **0.79*** |
| Alcohol abuse | 20.0% | 5.88% | .237 | 0.25 | 0.03 | 2.49 | 0.43 |
| Drugs abuse | 25.0% | 11.8% | .316 | 0.40 | 0.07 | 2.39 | 0.35 |
| Smoker | 60.0% | 66.7% | .819 | 1.33 | 0.11 | 15.70 | 0.14 |

**Table S2**: Distribution for the outcomes baed on the ED subtype and the presence of the DM.

|  | No-DM | | | | With T1DM | | | |
| --- | --- | --- | --- | --- | --- | --- | --- | --- |
|  | AN | BN | EDNOS | BED | AN | BN | EDNOS | BED |
| Drop-out | 0 (0%) | 0 (0%) | 4 (36.4%) | 1 (50.0%) | 0 (0%) | 2 (40.0%) | 7 (63.6%) | 1 (50.0%) |
| Partial or total remission | 2 (100%) | 5 (100%) | 7 (63.6%) | 1 (50.0%) | 2 (100%) | 3 (60.0%) | 4 (36.4%) | 1 (50.0%) |

AN: anorexia. BN: bulimia. EDNOS: eating disorder non-otherwise-specified. BED: binge eating disorder.

**Table S3**: Comparison for therapy outcomes between ED with and without T1DM

|  | Proportions | | Proportions comparisons | | | | |
| --- | --- | --- | --- | --- | --- | --- | --- |
|  | No-DM  (*n*= 20) | With T1DM.  (*n* = 20) | *p* | OR | 95% CI (OR) | | Cohen’s  | *d |* |
| Dropout (yes vs no) | 5 (25.0%) | 10 (50.0%) | .108 | 3.00 | 0.79 | 11.5 | **0.53*** |
| Partial or total remission | 15 (75.0%) | 10 (50.0%) | .108 | 0.33 | 0.09 | 1.27 | **0.53*** |

***Bold:** significant comparison (.05 level). *Bold: moderate to high effect size (|*d|*≥0.5).
